# Complete genome sequence of *Promicromonospora* sp. strain Populi*,* an actinobacterium isolated from *Populus trichocarpa* rhizosphere

**DOI:** 10.1128/mra.00851-24

**Published:** 2024-10-29

**Authors:** Mircea Podar, Leah H. Hochanadel, William G. Alexander, Christopher W. Schadt, Dale A. Pelletier

**Affiliations:** 1 Biosciences Division, Oak Ridge National Laboratory, Oak Ridge, Tennessee, USA; University of Strathclyde, Glasgow, United Kingdom

**Keywords:** rhizosphere, bacteria, symbiosis, plant-microbe interactions

## Abstract

*Promicromonospora* sp. strain Populi is an actinobacterium isolated from the rhizosphere of a black cottonwood tree, *Populus trichocarpa*. We completely sequenced its 5.2-Mbp chromosome using Oxford Nanopore long reads and predicted it to encode 4,685 proteins, 3 rRNA operons, and 54 tRNAs and noncoding RNAs.

## ANNOUNCEMENT

Members of the bacterial phylum Actinomycetota are abundant and diverse colonizers of plant rhizosphere and various tissues, many of them promoting growth and contributing to stress adaptation or resistance to pathogens (1–4). Among them, *Promicromonospora* species have been characterized from soils and the roots and leaves of a variety of plants and can confer drought tolerance and resistance to fungal diseases (5–11).

We describe the complete genome sequence of *Promicromonospora* sp. strain Populi, isolated from the rhizosphere of a mature, wild *Populus trichocarpa* from the Tieton River watershed in Washington State, USA (latitude: 46^o^42′11″ N, longitude: 120^o^39′37″ W). A rhizosphere sample (fine roots and adhering soil) was used to obtain a microbial fraction by centrifugation on Histodenz (Sigma-Aldrich Inc., St. Louis, MO, USA) (12) and stained with 5 µM Syto59 (Fisher Scientific Inc., Waltham, MA, USA). A Cytopeia Influx cell sorter (BD, Franklin Lakes, NJ, USA) was used to deposit individual single cells (100 per plate) based on forward-side scatter and fluorescence intensity on various nutrient agar media, as we described previously (13). Following incubation at 28°C for 5 days, we performed taxonomic characterization of individual bacterial colonies by amplification of the small subunit rRNA gene with universal primers (27F-1492R), followed by Sanger sequencing of the PCR product (Eurofin Genomics, Louisville, KY, USA). The sequence was compared to those of other organisms by blastn search of the NCBI rRNA sequence archive (13). A white, rugous colony from a plate with TWYE medium (DSMZ 1625) was identified as a *Promicromonospora* strain, based on >99% pairwise sequence identity to described species (*Promicromonospora alba,* NR_148779; *Promicromonospora kermanensis,* NR_156057). Therefore, we designated the isolate as *Promicromonospora* sp. strain Populi.

**Fig 1:**
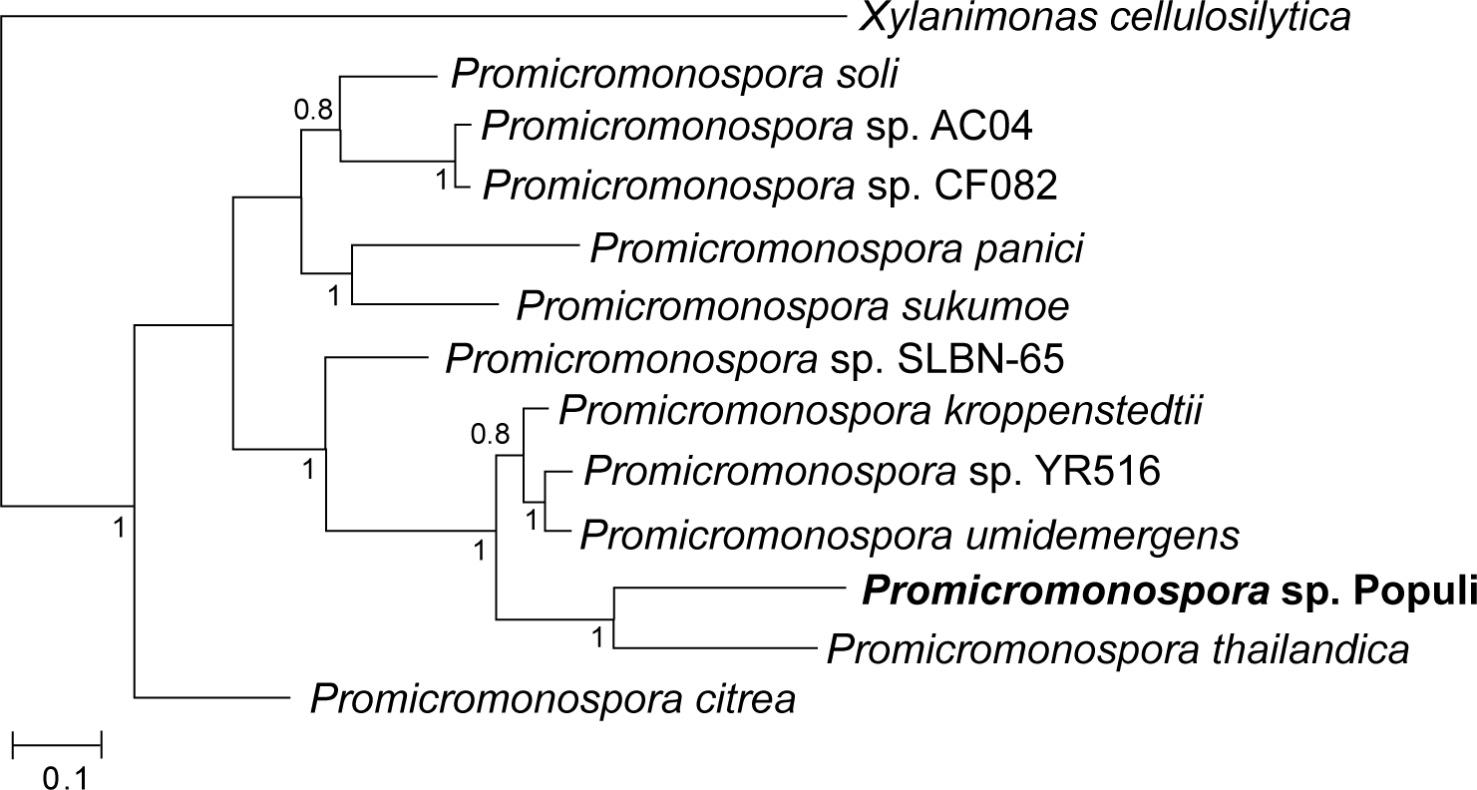
Maximum likelihood phylogenetic tree of *Promicromonospora* sp. strain Populi and related species, based on 49 core, universal bacterial proteins. The tree was computed using the default settings of SpeciesTree/FastTree2 in KBase, with estimated rates of evolution (CAT approximation) for each site. Numbers at the nodes indicate support values. The scale bar indicates estimated amino acid substitutions per site.

We grew the bacterium in 5 mL of liquid R2A medium for 3 days at 28°C and purified genomic DNA using the Qiagen DNeasy Blood and Tissue kit following the manufacturer’s recommended protocol (Qiagen, Germantown, MD, USA). A non-sheared, unselected library was prepared using the Oxford Nanopore Ligation Sequencing Kit followed by long-read sequencing on a MinION R10.4.1 (Oxford Nanopore Technologies Inc., Cambridge, MA, USA), yielding 379,306 reads (2.9 Gb) with an *N*_50_ of 7.6 kb. All data analyses were performed using software defaults. Base calling was performed using Dorado version 0.7.1 high-accuracy calling model. The reads were filtered to retain the top 1 Gbp of reads over 1 kb with Filtlong version 0.2.1, then used in the Trycycler version 0.5.4 workflow (14) together with Flye version 2.9.4 (15), Raven version 1.5.3 (16), and Miniasm/Minipolish version 0.3 (17). The final assembly was polished using Medaka version 1.5. The assembler output was a single circular contig, 5,224,924 bp in length, with 94-fold mean coverage and a G + C content of 70.9%. A protein phylogenetic tree using SpeciesTree version 2.2.0 (18) in KBase (19), using the default set of 49 core, universal bacterial genes places strain Populi closest to *Promicromonospora thailandica, Promicromonospora kroppenstedtii,* and *Promicromonospora umidemergens* as well as to other strains associated with *Populus deltoides* (AC04, CF082, and YR516) (13) (Fig. 1). Pairwise whole genome average nucleotide identity (ANI) among *Promicromonospora* species, including strain Populi, calculated using FastANI version 0.1.3 (20) ranged between 85% and 89% (Table 1). Gene prediction and functional annotation were performed using the NCBI PGAP version 6.7 (21).

**TABLE 1:** Pairwise ANI (%) between *Promicromonospora* genomes. GenBank accession numbers are indicated.

Genomes compared		ANI
*Promicromonospora sukumoe* GCF_014137995.1	*Promicromonospora* sp. Populi	86.3
*Promicromonospora kroppenstedtii* GCF_000515355.1	*Promicromonospora* sp. Populi	85.5
*Promicromonospora kroppenstedtii* GCF_000515355.1	*Promicromonospora sukumoe* GCF_014137995.1	89.7
*Promicromonospora* sp. AC04 GCF_003058225.1	*Promicromonospora* sp. Populi	88.1
*Promicromonospora citrea* GCF_014647735.1	*Promicromonospora* sp. Populi	84.1
*Promicromonospora* sp. AC04 GCF_003058225.1	*Promicromonospora citrea* GCF_014647735.1	84.6
*Promicromonospora thailandica* GCF_024171955.1	*Promicromonospora* sp. Populi	88.4
*Promicromonospora umidemergens* GCF_024171995.1	*Promicromonospora* sp. Populi	86.2
*Promicromonospora thailandica* GCF_024171955.1	*Promicromonospora* umidemergens GCF_024171995.1	87.0

## Data Availability

The *Promicromonospora* sp. strain Populi genome sequence has been deposited in GenBank under the accession number CP163528. The version described in this paper is the first version, CP163528.1. The sequence reads have been deposited in SRA under the accession number SRR30035506.
